# Post‐COVID‐19 condition: clinical phenotypes, pathophysiological mechanisms, pathology, and management strategies

**DOI:** 10.1002/path.6443

**Published:** 2025-06-10

**Authors:** Larissa E Vlaming‐van Eijk, Guolu Tang, Arno R Bourgonje, Wilfred F A den Dunnen, Jan‐Luuk Hillebrands, Harry van Goor

**Affiliations:** ^1^ Department of Pathology and Medical Biology, Division of Pathology University of Groningen, University Medical Centre Groningen Groningen The Netherlands; ^2^ Department of Gastroenterology and Hepatology University of Groningen, University Medical Centre Groningen Groningen The Netherlands; ^3^ The Henry D. Janowitz Division of Gastroenterology, Department of Medicine Icahn School of Medicine at Mount Sinai New York NY USA

**Keywords:** long COVID, post‐acute sequelae, clinical phenotypes, pathophysiology, management strategies

## Abstract

Post‐COVID‐19 condition (PCC), also known as long COVID, is a complex multiple organ system condition that can develop and persist for months after acute COVID‐19. PCC encompasses a wide range of symptoms, resulting in heterogeneous clinical manifestations. These manifestations likely arise from diverse underlying pathophysiological mechanisms, which, in turn, are influenced by risk factors such as age, sex, and comorbidities. To this end, characterising clinical phenotypes of PCC is essential for deepening our understanding of its (potentially) distinct pathophysiological mechanisms and for advancing diagnostic and patient‐tailored management strategies. PCC is thought to result from a complex interaction of various pathophysiological mechanisms, leading to functional and structural pathological alterations across multiple organ systems. Investigating these alterations is critical to improving our currently incomplete understanding of PCC's complex pathophysiology. This review provides an overview of the main clinical phenotypes of PCC, characterises these phenotypes by examining symptoms and signs, as well as the associated risk factors. The main hypothesised pathophysiological mechanisms are discussed by outlining the current knowledge on PCC pathology, focussing on the most commonly affected organ systems. Current PCC management includes supportive care such as physiotherapy and the repurposing of existing drugs primarily targeting persistence of SARS‐CoV‐2 (e.g. antivirals, monoclonal antibodies) and immune dysfunction (e.g. antiinflammatory drugs, immunomodulators). To date, prevention of SARS‐CoV‐2 infection remains critical, which can be achieved through effective public health measures and vaccination strategies. Finally, this review highlights current knowledge gaps and proposes future research directions to advance the understanding and treatment of PCC. © 2025 The Author(s). *The Journal of Pathology* published by John Wiley & Sons Ltd on behalf of The Pathological Society of Great Britain and Ireland.

## Introduction

Coronavirus disease 2019 (COVID‐19), driven by severe acute respiratory syndrome coronavirus 2 (SARS‐CoV‐2), has had a lasting impact on human well‐being and global healthcare over the past years. Although significant strides have been made to combat acute COVID‐19, now considered endemic, some survivors go on to develop lingering symptoms long after acute infection, a condition widely known as ‘Post‐COVID‐19 condition’ (PCC) or ‘long COVID’. Other common terms used to describe long‐term health effects of COVID‐19 include ‘post‐acute sequelae of SARS‐CoV‐2 (PASC)’, ‘post‐acute COVID‐19 syndrome (PCS)’, or ‘long haulers syndrome’ [1, 2]. The definitions of PCC are broad, complicating efforts to capture the diverse manifestations of the condition comprehensively. Referring to specific syndromes rather than the general term PCC would likely improve its understandibility. The World Health Organization adopted the term PCC to describe long‐term symptoms persisting or newly emerging at least 3 months following acute SARS‐CoV‐2 infection that continue for at least 2 months without any other identifiable cause [3]. The condition is a clinical diagnosis due to the lack of definitive laboratory tests or established biomarkers.

PCC can present with a wide range of symptoms – over 200 have been identified – that are multiple organ system‐specific, affecting, among others, the nervous, respiratory, cardiovascular, musculoskeletal, gastrointestinal, and urinary organ systems. Common clinical symptoms include dyspnoea, fatigue, and cognitive dysfunction (also known as ‘brain fog’). These symptoms can fluctuate over time, but often disrupt daily life. Individuals particularly at risk of developing PCC include the elderly, females, those who are not (or insufficiently) vaccinated, individuals who experienced severe acute COVID‐19 requiring hospitalisation, and those with (multiple) comorbidities such as cardiovascular disease, chronic pulmonary disease, and obesity [4, 5]. These comorbidities can, individually or in combination, exacerbate or be exacerbated by PCC [6, 7]. However, PCC can affect individuals of all ages, genders, and ethnicities, including those who were previously healthy, vaccinated, and had only mild initial SARS‐CoV‐2 infections [4]. In fact, since the majority of individuals with acute COVID‐19 were not hospitalised, most people with PCC had only mild initial illness [8]. The reasons why some people fully recover from COVID‐19 while others develop PCC remain unclear.

Defining symptom‐based PCC phenotypes and their associated risk factors may help us understand the underlying pathophysiological mechanisms of the disease. It is unlikely that a single pathway accounts for the development and progression of this complex condition, and a number of different pathways are probably involved. These interconnected pathways may reinforce one another, creating vicious cycles of ongoing pathology that result in lasting tissue injury and dysregulated tissue repair. To this end, dissecting the main pathological alterations (both on a functional and structural level) observed in the most commonly affected organ systems is crucial, not only for deepening our understanding of its underlying pathophysiology, but also for advancing diagnostic and patient‐tailored treatment strategies.

This review aims to identify and categorise the main clinical phenotypes of PCC, examining their associated risk factors and the potential underlying pathophysiological mechanisms by outlining the functional and structural pathology. In addition, the rationale for various treatment modalities tailored to these phenotypes is discussed. Finally, we highlight areas where current knowledge of PCC remains incomplete and propose directions for future research to address these gaps.

## Putative pathophysiological mechanisms underlying PCC

**Figure 1:**
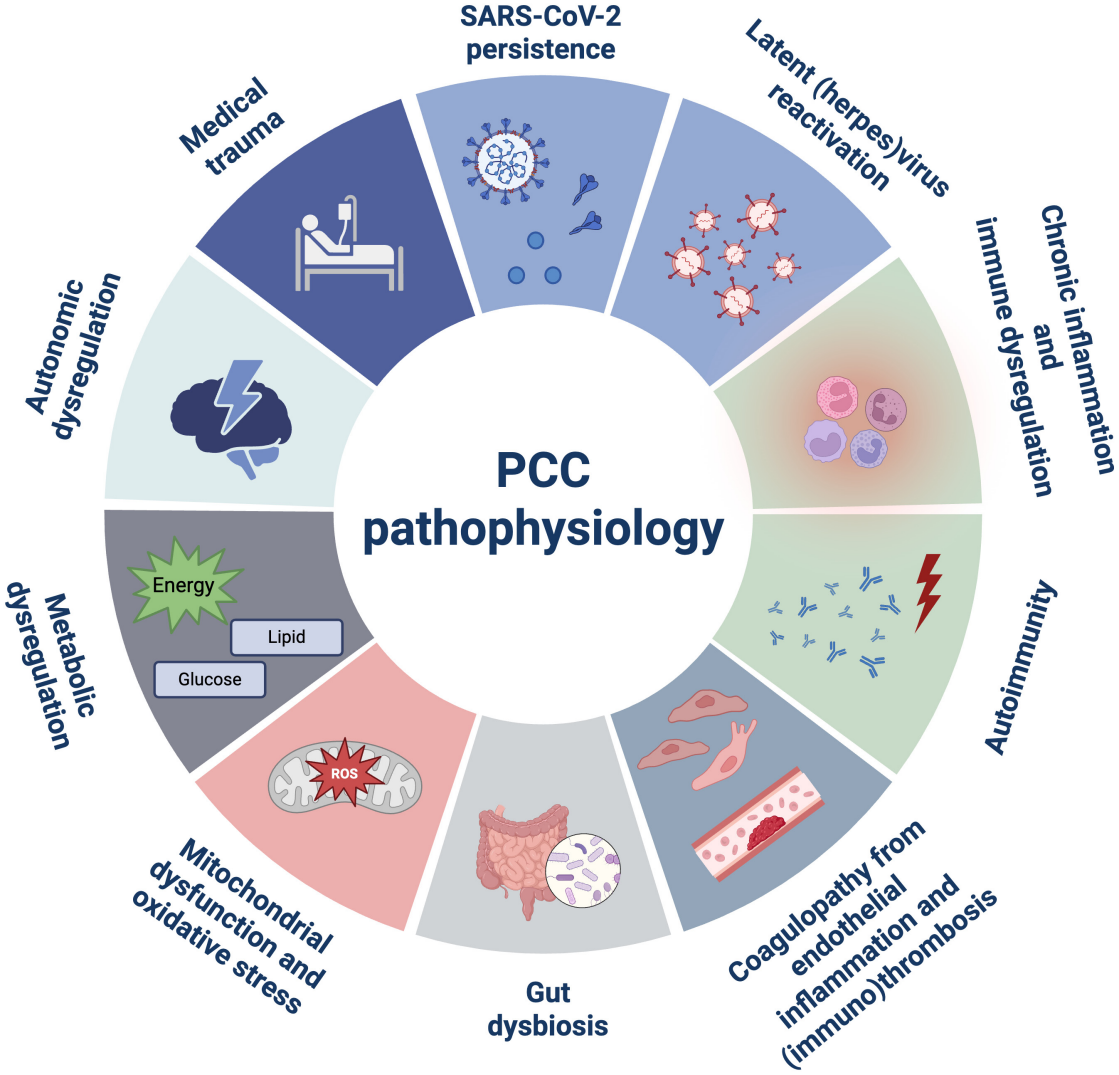
Hypothesised pathophysiological pathways underlying PCC. Hypothesised pathways include virus‐related mechanisms, including viral persistence in tissues (either SARS‐CoV‐2 itself or its components) and latent virus reactivation (such as Epstein–Barr virus or other herpesviruses), chronic inflammation and immune dysregulation, autoimmunity, coagulopathy from endothelial inflammation and (immuno)thrombosis, gut dysbiosis, mitochondrial dysfunction and oxidative stress, metabolic dysregulation, autonomic dysregulation, and long‐term sequelae of medical trauma (e.g. postintensive care syndrome and posttraumatic stress disorder). This figure was created using BioRender (https://BioRender.com).

The development of PCC is thought to occur as a result of a complex interplay of multiple pathophysiological pathways (Figure 1). These include: (1) virus‐related mechanisms including viral persistence in tissues (either SARS‐CoV‐2 itself or its components, such as spike and nucleocapsid proteins after incomplete viral clearance) and latent virus reactivation (such as Epstein–Barr virus or other herpesviruses); (2) chronic inflammation and immune dysregulation; (3) autoimmunity; (4) coagulopathy from endothelial inflammation and (immuno)thrombosis; (5) gut dysbiosis; (6) mitochondrial dysfunction and oxidative stress; (7) metabolic dysregulation; (8) autonomic dysregulation; and (9) long‐term sequelae of medical trauma (e.g. postintensive care syndrome and posttraumatic stress disorder) [8, 9]. Depending on individual risk factors, these pathophysiological pathways may orchestrate the heterogeneous clinical manifestations of PCC, discussed in more detail below.

## Clinical characterisation and risk factors

**Figure 2:**
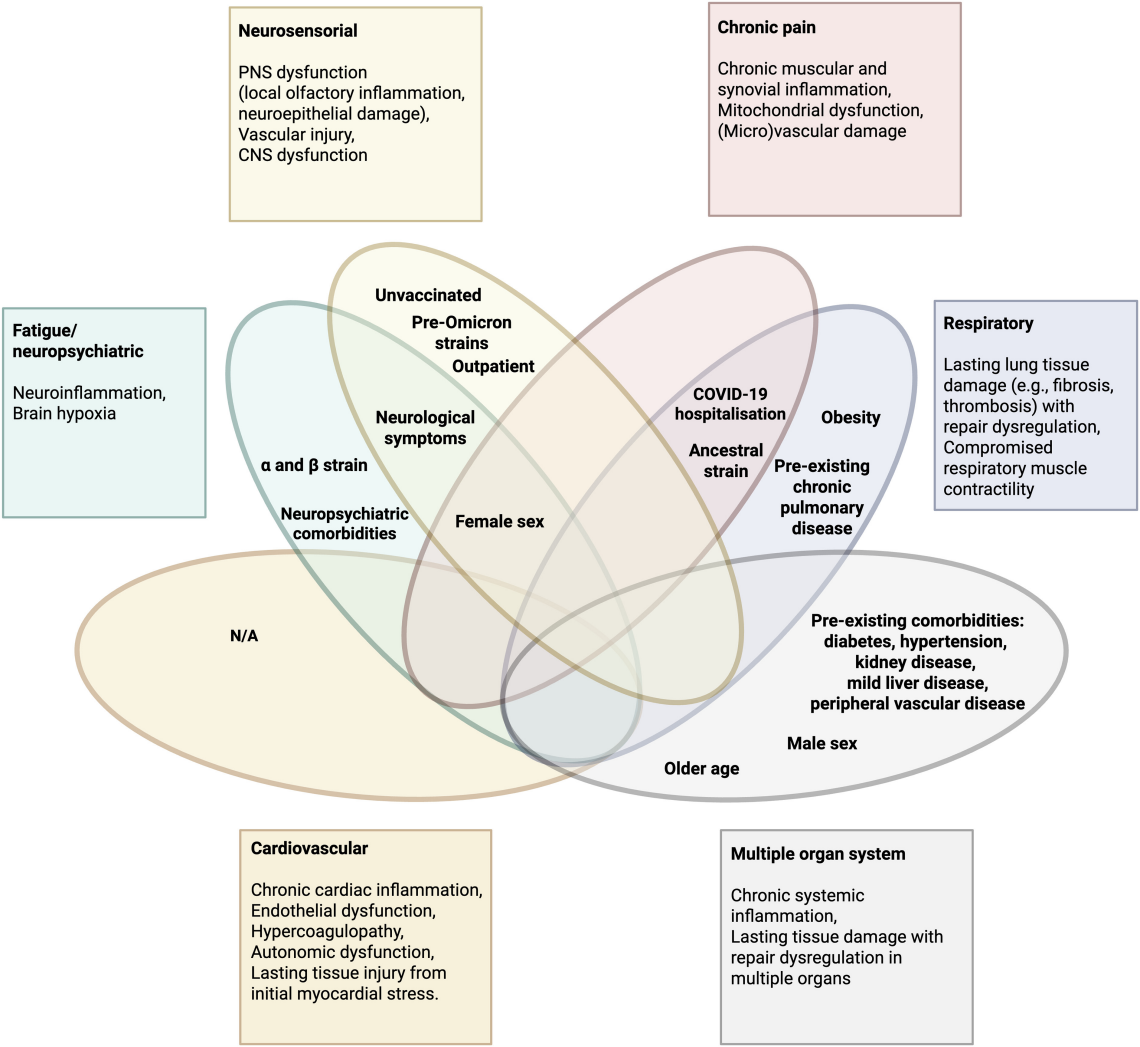
Symptom‐based clinical phenotypes of PCC, their associated risk factors, and putative underlying pathophysiological mechanisms. Risk factors are represented within the oval shaded areas, whereas the putative underlying pathophysiological mechanisms are outlined in the correspondingly shaded boxes. Abbreviations: CNS, central nervous system; COVID‐19, coronavirus disease 2019; N/A, not applicable; PNS, peripheral nervous system; PCC, Post‐COVID‐19 Condition; SARS‐CoV‐2, severe acute respiratory syndrome coronavirus 2. This figure was created using BioRender (https://BioRender.com).

Given the heterogeneity of PCC symptoms, identifying symptom clusters and linking them to specific risk factors and potential biological mechanisms could lead to more targeted treatment and care. Previous evidence from machine‐learning approaches supports the existence of distinct PCC phenotypes [10, 11, 12, 13, 14, 15]. However, their characterisation remains inconsistent and is highly dependent on study group characteristics (e.g. hospitalisation status, SARS‐CoV‐2 variant, different timepoints post‐COVID‐19) and the methods used (e.g. principal component analysis, multiple correspondence analysis, semantic similarity, k‐means clustering, hierarchical clustering, among others) [10, 11, 12, 13, 14, 15]. Despite these differences, these studies reveal several recurring symptom clusters. Considering their frequent emergence across multiple studies, despite variations in study design and methodology, these phenotypes were selected as a framework for categorising PCC symptoms in this review (Figure 2). These include fatigue/neuropsychiatric, neurosensorial, respiratory, chronic pain, cardiovascular, and multiple organ system phenotypes [10, 11, 12, 13, 14, 15]. However, it must be noted that the clinical presentation of PCC varies, ranging from well‐defined and easily recognisable phenotypes to complex, multiple organ system manifestations. For some symptoms (e.g. fatigue, postexertional malaise) there was considerable overlap between clusters, supporting the existence of multiple pathophysiological processes producing overlapping phenotypes.

### Fatigue/neuropsychiatric phenotype

A cognitive or neuropsychiatric PCC phenotype is identified by symptoms such as brain fog (including poor concentration, memory loss, and difficulty in thinking), insomnia, anxiety, and depression. Multiple cluster analysis studies combined this phenotype with chronic fatigue [10, 11, 15], although it must be mentioned that fatigue is a common symptom in multiple phenotypes, as it is a broad concept (e.g. physical, mental, or emotional fatigue) with multiple underlying pathophysiological pathways. Sleep disturbance including insomnia is another common symptom, which by itself may lead to cognitive difficulties and emotional distress.

The fatigue/neuropsychiatric phenotype is the most common in the literature, representing 42% of PCC individuals in one study [10]. Neurological involvement in PCC has also become evident in societal contexts, manifesting in cognitive difficulties with work, social participation, and daily functioning. This phenotype was the most common phenotype after alpha and delta SARS‐CoV‐2 variants, whereas a cluster of cardiorespiratory symptoms was the most common in the ancestral variant, possibly indicating lung damage [15]. Furthermore, this phenotype was more prevalent in females than males, and had a high proportion of preexisting depression in one study [13], highlighting the possibility of preexisting neuropsychiatric comorbidities as a risk factor. Preexisting neurological and psychiatric conditions may compromise the central nervous system's resilience to manage additional stressors, including viral infections, leading to a higher risk of persistent symptoms such as brain fog, fatigue, and mood disturbances [16, 17]. A French population‐based study demonstrated that the higher prevalence of PCC in women is partially explained by their greater burden of depressive symptoms during the pandemic, as depressive symptoms contributed to around 40–45% of the observed sex differences [18]. This highlights that taking both mental health factors and biological sex differences into account is essential for understanding and managing the fatigue/neuropsychiatric manifestations of PCC. Sleep disorders in PCC, particularly insomnia, are more commonly observed in females, with a suggested relation to affective disorders such as depression or anxiety, whereas men primarily experience sleep‐related issues including snoring and sudden movements, mostly associated with respiratory disorders [19, 20]. Finally, the fatigue/neuropsychiatric phenotype was associated with experiencing neurological symptoms during acute COVID‐19 diagnosis [10], suggesting that the driver of this phenotype may be ongoing neuropathology induced by acute COVID‐19, rather than a distinct, new event.

### Neurosensorial phenotype

This phenotype is characterised by alterations in the sense of smell and/or taste, which are hallmark symptoms of both acute COVID‐19 and PCC, representing some of the most lingering and consistent manifestations of the disease. Numerous studies have demonstrated the significance of olfactory and gustatory dysfunctions in the context of SARS‐CoV‐2 infection. For example, an experimental study involving direct exposure to SARS‐CoV‐2 revealed that two‐thirds of infected individuals experienced varying degrees of smell and taste dysfunction over 6 months [21]. Furthermore, individuals with anosmia or ageusia showed a 17 times higher odds of testing positive for COVID‐19 compared to those without these symptoms [21]. However, reported prevalence rates for anosmia and ageusia in COVID‐19 vary widely, ranging from 8% to 85%, reflecting factors such as age, sex, race, genetics, and vaccination status [21]. Alterations in smell and/or taste may last after acute COVID‐19, with this phenotype representing 11% of participants with PCC in one study [10]. Interestingly, prolonged symptoms of alterations in smell and/or taste were more commonly observed earlier in the pandemic, whereas these symptoms are less commonly observed with vaccination and omicron infection [11]. Other risk factors include female sex and not being hospitalised during acute COVID‐19 [10, 11]. A large meta‐analysis in PCC patients after both hospitalised and nonhospitalised COVID‐19 demonstrated pooled prevalence rates of loss of sense of smell and taste of 6.3% and 5.4% in hospitalised vs. 12.7% and 8.7% in nonhospitalised individuals [22], respectively. Besides biological host‐specific factors (e.g. receptor status, differences in local inflammatory response), potential explanations include inflammation‐reducing treatments in hospitalised patients and less focus on relatively innocent symptoms such as anosmia or ageusia in severe cases, leading to these symptoms going unnoticed or unreported. Again, neurological symptoms at initial SARS‐CoV‐2 diagnosis were associated with the neurosensorial phenotype, suggesting lasting injury as an explanation for these symptoms (see section ‘Pathology and underlying pathophysiological mechanisms’ below).

### Respiratory phenotype

The respiratory PCC phenotype is characterised primarily by cough and dyspnoea, with hypoxaemia frequently observed [13]. The risk of respiratory symptoms is highest 30–90 days after infection and tends to decrease over time [23]. Nevertheless, lingering pulmonary sequelae can cause significant morbidity and disability [23]. Compared to other phenotypes, the respiratory phenotype has been associated with a particularly high reduction in quality of life [10]. In one study, the respiratory phenotype was the largest phenotype in the period dominated by the wildtype (ancestral) SARS‐CoV‐2 strain when the population was unvaccinated [15]. Other risk factors include obesity and hospitalisation for acute COVID‐19, independent of potential confounders such as sex, age, race/ethnicity, comorbidities, or vaccination status [11]. Hospitalisation being an independent risk factor for prolonged respiratory complaints within this phenotype suggests that these symptoms may be attributed to lasting lung injury due to severe acute COVID‐19 or its hospital treatment. One study reported that preexisting chronic pulmonary disease was a risk factor for developing lasting respiratory symptoms [10], although another study observed similar rates in other phenotypes, including neuropsychiatric and cardiovascular PCC clusters [13].

### Cardiovascular phenotype

Symptoms such as tachycardia, palpitations and chest pain dominate the cardiovascular phenotype, with hypoxaemia frequently observed [13]. Patients with preexisting cardiovascular disease are at a higher risk of developing PCC in general [4, 5]. It has been hypothesised that this occurs as a result of acute COVID‐19 worsening preexisting cardiovascular conditions, either directly from COVID‐19‐related effects on the cardiovascular system or indirectly through cardiovascular side effects of COVID‐19‐related drugs (such as altered cardiac conduction with antivirals, or hypertension with interleukin blockers), or reduced access to cardiovascular interventions during the pandemic [24]. However, no specific risk factors have been identified by cluster analysis, suggesting that these types of symptoms may occur in cardiovascular‐burdened and previously healthy individuals. In particular, preexisting cardiovascular diseases (heart failure, coronary artery disease, myocardial infarction, cardiomyopathies) were not overrepresented in the cardiovascular PCC phenotype in comparison to other phenotypes [13]. This finding contradicts the hypothesis that this phenotype develops due to acute COVID‐19 worsening preexisting cardiovascular conditions, although this may still hold true for subclinical cardiovascular disease [25]. However, one would still expect an increased cardiovascular risk profile, yet factors such as hypertension, tobacco smoking, and diabetes mellitus were not significantly overrepresented in the cardiovascular PCC phenotype [13]. Instead, cardiovascular symptoms in PCC are likely to affect both cardiovascular‐burdened and previously healthy individuals, suggesting a new COVID‐19‐specific phenomenon impacting cardiovascular health beyond traditional risk factors, which will be further explored below (‘Pathology and underlying pathophysiological mechanisms’).

### Chronic pain phenotype

The chronic pain phenotype is characterised by symptoms such as arthralgia and myalgia and is marked by a high reduction in quality of life [10]. Female patients are especially at risk of developing chronic musculoskeletal pain. Other risk factors include hospitalisation for acute COVID‐19 [22, 26] and infection by the ancestral SARS‐CoV‐2 variant, independent of vaccination [27]. New‐onset chronic musculoskeletal pain in individuals with PCC often fulfil the diagnostic criteria for fibromyalgia, indicating potential similar underlying mechanisms [28].

### Multiple organ system phenotype

The above‐described phenotypes are not always clear‐cut and some individuals present with complex multiple organ system manifestations [12, 13, 14, 15] and multiple studies report a separate multiple organ system phenotype [12, 13, 15]. An interesting observation from a large cluster analysis of hospitalised (inpatients) and those not requiring hospitalisation (outpatients) with PCC was a multiple organ system cluster characterised by high‐frequency laboratory abnormalities associated with a severe course of acute COVID‐19, such as lymphopenia, thrombocytopaenia, elevated levels of alanine aminotransferase, alkaline phosphatase, and ferritin [13]. This phenotype contained statistically higher proportions of inpatients (34.0%) compared to any other cluster (15.2–21.0%), with a higher prevalence of acute kidney injury (AKI) and corticosteroid use during acute COVID‐19, indicating that this phenotype may represent individuals experiencing residual effects from more severe COVID‐19. The study suggests that older age, male sex, and certain comorbidities (diabetes, hypertension, kidney disease, mild liver disease, and peripheral vascular disease) are risk factors for this severe multiple organ system phenotype. However, less severe multiple organ system phenotypes with only a few or mild PCC symptoms and less functional impairment have also been identified [13, 14].

### Other common symptoms and risk factors

Besides the above‐described clinical presentations of PCC, other symptoms and risk factors have been identified. Examples of frequently occurring symptoms that clustered in multiple phenotypes are muscle fatigue or postexertional malaise [15] and gastrointestinal symptoms such as abdominal pain, nausea, and diarrhoea [12], which will be elaborated on in the next section (‘Pathology and underlying pathophysiological mechanisms’). In addition, several risk factors for developing PCC in general have been identified that may not pose a risk to a particular PCC phenotype.

While males are more susceptible to severe acute COVID‐19, women are disproportionally affected by PCC, a pattern that remains incompletely understood. It is thought that both psychosocial factors (e.g. mental health conditions including depression) and biological factors (e.g. hormonal factors, differences in immune response, autoimmune predisposition) (*vide infra*) account for the observed sex differences, underscoring a biopsychosocial model as an essential approach for understanding and managing sex‐related manifestations in PCC [18, 29].

Metabolic disorders, particularly obesity and diabetes, have emerged as significant risk factors for both severe COVID‐19 and PCC [4, 5, 30]. A population‐based longitudinal prospective study highlighted that individuals with preexisting diabetes (type unknown) show a 37% increased risk to develop PCC compared to nondiabetic individuals [31]. Another study demonstrated that type 2 diabetes (and not type 1 diabetes) was a predisposing factor, but only when body mass index (BMI) was not included in the analysis, indicating that BMI may be an important confounder in the association [32]. Preliminary results showed no convincing evidence for associations of HbA1c with PCC, suggesting that obesity‐associated mechanisms may be responsible for increased PCC risk in diabetes [33].

Autoimmune diseases, such as rheumatoid arthritis (RA), may also increase the risk of PCC [34, 35]. Autoimmunity is more prevalent in middle‐aged women—those at greater risk for PCC [4]. These conditions are characterised by an overactive immune response that targets the body's own tissues, leading to chronic inflammation and tissue damage. When combined with the inflammatory response to SARS‐CoV‐2, this preexisting immune dysregulation may result in a severe and prolonged disease course. Moreover, immunosuppressive treatments, often used to manage autoimmune diseases, may result in prolonged viral shedding and predispose to secondary infections or reactivation of latent viruses, complicating recovery [36, 37]. However, it should be noted that classifying patients with autoimmune diseases as having PCC is challenging due to overlapping symptoms, and preexisting autoimmune disease as a risk factor for PCC is not yet certain [34, 38].

While substantial evidence suggests a link between genetic predisposition and acute COVID‐19, less is understood about the genetic factors influencing PCC. Genome‐wide association studies have identified potential variants associated with PCC, although none have reached statistical significance [39, 40, 41]. Epigenetics are likely to play a role, with preliminary results indicating persisting changes in DNA methylation 1 year after acute COVID‐19 [42]. Ongoing research aims to uncover more definitive associations and understand how they may modulate the immune response, inflammation, or tissue repair mechanisms in the context of PCC [43].

## Pathology and underlying pathophysiological mechanisms

**Figure 3:**
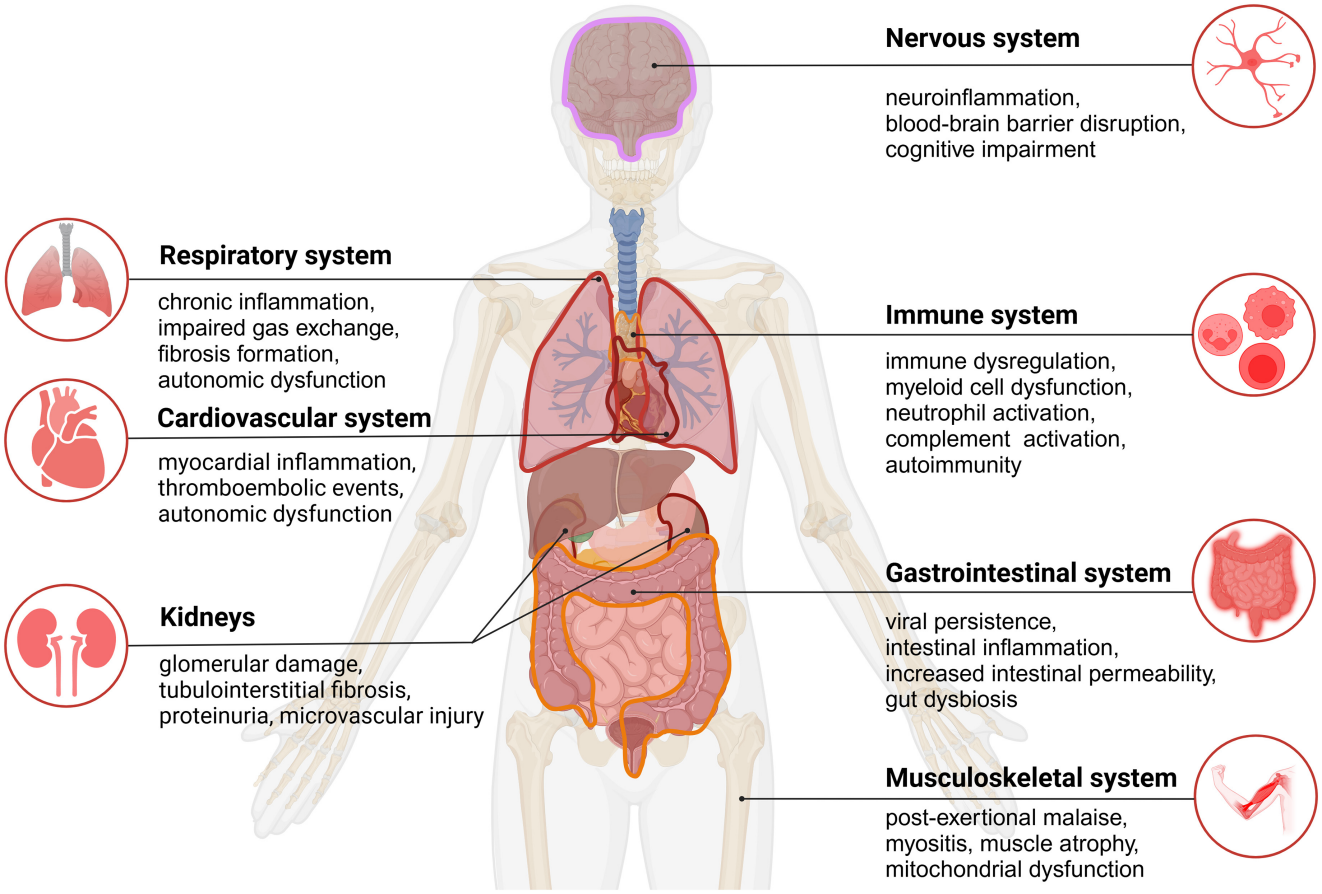
Multiple organ system pathophysiological manifestations of PCC. This illustration highlights the main organ systems affected in PCC, including the respiratory, cardiovascular, nervous, immune, gastrointestinal, and musculoskeletal systems, each with distinct pathological changes contributing to the broad spectrum of clinical symptoms. This figure was created using BioRender (https://BioRender.com).

PCC can involve a diverse set of organ systems and understanding the mechanisms of injury is pivotal to enhance our understanding of PCC pathophysiology (Figure 3). Knowledge on pathology in PCC is currently limited and complicated by confounding factors such as comorbidities (see section ‘Future directions and research gaps’, below). Nevertheless, research studies such as RECOVER‐Pathology—an autopsy study investigating PCC in adults—are ongoing [44] and are beginning to clarify the pathological changes observed in these patients. Notably, plasma proteomic profiling highlights distinct biomarker signatures associated with different PCC phenotypes, which may enhance our understanding of the underlying pathophysiology [45, 46]. The main pathological alterations (on the functional and structural levels) in the most commonly affected organ systems are discussed in the following paragraphs.

### Nervous system

#### Fatigue/neuropsychiatric PCC phenotype

Neurological symptoms during acute COVID‐19 correlate with developing the fatigue/neuropsychiatric and neurosensorial phenotypes of PCC, suggesting that COVID‐19‐induced neuropathology lingers after initial infection resolution [10]. Indeed, neuroimaging studies have revealed structural brain changes in the postacute phase of COVID‐19, correlating with prolonged neurological and neuropsychiatric symptoms [47, 48, 49, 50]. For instance, reductions in grey matter thickness in the olfactory cortex and limbic system have been observed in scans taken before and 4–5 months postinfection, even in mild cases [47]. Changes in shape and reduced volumes of the thalamus and basal ganglia have been linked to post‐COVID fatigue and short‐term memory problems [48], while hippocampal alterations may contribute to memory and cognitive dysfunctions in PCC [51].

Ongoing neuroinflammation is a prominent hypothesised driver of the fatigue/neuropsychiatric phenotype. Autopsy studies of COVID‐19 patients have shown signs of neuroinflammation, including widespread activation of microglia and astrocytes, particularly in the brainstem [52, 53]. Neuroinflammation in fatal COVID‐19 cases suggests that ongoing neuropathology may be present in PCC patients. In agreement, positron emission tomography (PET) neuroimaging showed evidence of neuroinflammation across multiple brain areas (including the cingulate cortex, corpus callosum, thalamus, basal ganglia, and circumventricular organs) in individuals with PCC compared to healthy controls [54, 55]. Of note, these findings were linked to persistent depressive and cognitive symptoms [55]. In support of this, a proteomic study in PCC patients hospitalised for acute COVID‐19 found plasma signatures suggestive of neuroinflammation (C1QA and DPP10) and dysfunctional nerve tissue repair (SPON‐1 and NFASC) in a subset of PCC patients with cognitive symptoms [45]. These processes potentially result in neurodegeneration. Previous studies linked persistent viral infections such as herpes simplex virus‐1 (HSV‐1) with neuroinflammation and/or protein misfolding as a potential driver of cognitive decline and neurodegenerative diseases [56, 57]. HSV‐1 can infect peripheral sensory neurons and establish latent infection in the trigeminal ganglia, and may travel to the trigeminal nuclei, thalamus, and sensory cortex upon reactivation, causing encephalitis or persistent latent infection in the central nervous system (CNS) [56, 57]. Persistent infection and reactivation of HSV‐1 is associated with amyloid‐β plaque accumulation and tau protein hyperphosphorylation, both hallmarks of Alzheimer's pathology [56, 57]. Beyond direct neuropathology, HSV‐1 persistence is hypothesised to contribute to chronic neuroinflammation and oxidative stress, potentially triggering neurodegenerative disease in genetically predisposed individuals [56, 57]. While similar mechanisms have been proposed for SARS‐CoV‐2, they remain to be investigated using appropriate control groups (e.g. sepsis, alpha‐synucleinopathy, tauopathy, and age‐matched subjects who died from nonneurological‐related causes). Additionally, neuroinflammation in the brainstem may disrupt autonomic control, thereby contributing to the respiratory and cardiovascular PCC phenotypes, although more research is needed to confirm this link [58].

Why SARS‐CoV‐2 infection leads to neuroinflammation remains incompletely understood. Although direct CNS invasion by SARS‐CoV‐2 is possible, as viral RNA and proteins have been detected in brain tissue, the severity of neuropathological changes does not always correlate with viral presence [53, 59]. Instead, it seems more plausible that neuroinflammation results indirectly from systemic inflammation and disruption of the blood–brain barrier (BBB). This is supported by findings of vascular damage and perivascular inflammation in the brains of acute COVID‐19 patients [60]. In PCC, recent studies provide convincing evidence that a disrupted BBB in tandem with systemic hyperinflammation is a key driver of PCC‐associated cognitive impairment [54, 61]. PET neuroimaging demonstrated neuroinflammation in circumventricular organs, regions associated with reduced BBB integrity [54]. Other areas with an intact BBB were also observed to exhibit neuroinflammation, with activation of the neurovascular endothelial lining by circulating factors potentially driving glial activation, a theory supported by associations between neuroinflammation and vascular dysfunction markers (e.g. fibrinogen, sL‐selectin) [54]. Another study found that brain fog in PCC is associated with increased BBB permeability on dynamic contrast‐enhanced magnetic resonance imaging (DCE‐MRI), particularly in the temporal lobes and frontal cortex [61]. This increased BBB permeability was accompanied by structural brain changes (including reduced global brain volume, white matter volume, and cerebral volume, and increased cerebrospinal fluid volume) and associated with TGF‐β, indicating a potential role of immunovascular dysfunction in the pathophysiology of cognitive impairment [61].

Another hypothesised mechanism underlying the fatigue/neuropsychiatric PCC phenotype includes hypoxia‐induced damage. Early autopsy studies reported hypoxia‐related injury with neuronal loss in the brains of acute COVID‐19 patients [53, 62]. This damage may result from respiratory failure or vascular damage due to either a systemic pro‐thrombotic state with resulting cerebral (micro)thrombosis, or to local microvascular injury from endothelial dysfunction (virally‐induced or systemic hyperinflammation‐related) [9]. In keeping with this, coagulation abnormalities, including fibrinogen and D‐dimer levels during or shortly after acute COVID‐19, are associated with cognitive impairment in PCC [11, 63]. Lasting microvascular damage with resulting brain tissue hypoxia could underlie neurological symptoms in some individuals. Additionally, hypoxia‐induced damage may lead to oxidative stress and overproduction of reactive oxygen species (ROS), which can exacerbate neuronal damage and inflammation, potentially contributing to persistent symptoms in PCC [64]. Indeed, examining brain hypoxia post‐COVID‐19 using the novel technology frequency‐domain near‐infrared spectroscopy reported that 24% of individuals with mild COVID‐19 had signs of brain hypoxia at least 8 weeks after acute infection, which was associated with fatigue and depression despite normal systemic oxygenation [65]. Interestingly, hyperbaric oxygen therapy may be beneficial by triggering brain neuroplasticity, leading to improvements in long‐term PCC symptoms including neuropsychiatric symptoms and pain [66].

#### Neurosensorial PCC phenotype

In PCC, smell and taste dysfunctions persist as a hallmark phenotype, with pathophysiological mechanisms involving both the peripheral and the CNS. Several hypotheses have been proposed to explain the mechanisms underlying this phenomenon. One is local inflammation and blockage of odorant transit. Inflammation in the olfactory cleft, the narrow anatomical region above the turbinates where odorant molecules access olfactory receptors, may obstruct transmission of odorants to receptor cells. Imaging studies have consistently shown olfactory cleft obstruction in patients with anosmia early in the course of COVID‐19, with inflammation often resolving within weeks [67]. Inflammation in this region is often localised, potentially due to differential aerosol deposition during inhalation [68], and may explain why many patients with anosmia do not report nasal congestion or other respiratory symptoms [69]. Nasal fluids contain increased cytokines post‐COVID compared to SARS‐CoV‐2‐negative controls, but this was not associated with PCC symptoms [45], making a direct link unclear.

Another potential mechanism underlying the neurosensorial phenotype of PCC is damage to the olfactory neuroepithelium. Although olfactory receptor neurons do not express ACE2, SARS‐CoV‐2 infects the olfactory neuroepithelium via nonneuronal sustentacular and basal cells that express ACE2 receptors. Damage to these cells disrupts their supportive roles, indirectly impairing olfactory receptor function [70]. This may also be an explanation for why the neurosensorial phenotype is less common after infection with postancestral SARS‐CoV‐2 variants, as mutations in these variants reduce their efficiency in infecting TMPRSS2‐expressing host cells. Given that supporting cells in the olfactory epithelium abundantly express TMPRSS2, these cells are likely less susceptible to infection by postancestral variants, preserving olfactory function [71]. Additionally, SARS‐CoV‐2 infection indirectly impairs olfactory sensory neurons through noncell‐autonomous effects, mediated by disruptions in the surrounding supportive cells. These effects include altered nuclear architecture and downregulation of olfactory receptor gene expression, leading to persistent dysfunction [72]. Autopsied olfactory epithelia show reduced olfactory receptor protein levels and signalling components, highlighting persistent dysfunction. Key genes such as *ADCY3* and other olfactory receptor signalling pathways were downregulated in both hamsters and humans after SARS‐CoV‐2 inoculation [72].

Vascular and neurological injury may also play a role in neurosensorial symptoms in PCC. Interestingly, elevated plasma ICAM‐1 (a marker of endothelial activation) in the early postinfection period associates with a greater likelihood of developing the neurosensorial PCC phenotype [11]. SARS‐CoV‐2‐induced microangiopathy and endothelial dysfunction can impair blood flow to neurons involved in olfactory perception, potentially causing ischaemic damage to the olfactory bulb and related structures. Vascular injury from systemic inflammation and cytokine storms may disrupt the BBB, allowing inflammatory mediators to enter the CNS [73]. Haematogenous viral spread to BBB endothelial cells could damage pericytes and astrocytes, thereby affecting brain homeostasis and contributing to both central and peripheral olfactory disturbances [74].

Finally, central neurological mechanisms may be involved in the persistence of smell and taste dysfunction. Neuroimaging shows reduced olfactory bulb volumes and structural changes in central olfactory pathways in PCC patients, indicating CNS involvement. For instance, reductions in grey matter thickness in the olfactory cortex associate with persistent anosmia [47]. These findings suggest that central processes, in addition to peripheral damage, contribute to the persistence of smell and taste dysfunction [75].

### Respiratory system

While acute COVID‐19 primarily affects the respiratory system, only a subset of PCC patients experience respiratory issues [30]. Hospitalisation during acute COVID‐19 was found to be an independent risk factor for developing the respiratory PCC phenotype [11], suggesting that lasting lung tissue damage from severe acute COVID‐19 or its hospital treatment, accompanied by repair dysregulation, is a key mechanism underlying prolonged respiratory complaints. Besides hospitalisation, another risk factor is preexisting pulmonary disease [10]. Patients with chronic respiratory diseases such as chronic obstructive pulmonary disease (COPD) and asthma often face compromised respiratory muscle contractility and diminished lung function due to persistent airway inflammation, remodelling, and hyperresponsiveness [76]. In COPD, chronic exposure to irritants leads to fibrosis and smooth muscle hypertrophy that impair lung elasticity and muscle function [77]. Asthma is marked by chronic airway inflammation, leading to bronchoconstriction and airway hyperreactivity [78]. These alterations result in reduced inspiratory and expiratory muscle strength, making it more difficult for the lungs to maintain proper ventilation [79]. Following SARS‐CoV‐2 infection, these preexisting impairments may worsen due to additional inflammatory processes and COVID‐19‐related damage, leading to further reductions in long‐term pulmonary function [80]. Another specific risk factor for the PCC respiratory phenotype is obesity, which may be explained by the already impaired lung mechanics in obese patients, characterised by limited lung expansion and volume, as well as increased airway resistance. Other hypothesised mechanisms include chronic low‐grade inflammation and a potential viral reservoir in adipose tissue, albeit this likely causes systemic rather than respiratory system‐specific chronic symptoms [81, 82, 83]. Of note, obesity is also an established risk factor for severe acute COVID‐19, which in turn is associated with respiratory PCC, potentially leaving more lasting damage in lung tissue following acute COVID‐19.

During acute infection, SARS‐CoV‐2 invades the respiratory tract via epithelial and alveolar type 2 cells that express the ACE2 receptor required for viral entry [84, 85]. In some individuals, inadequate initial immune responses lead to uncontrolled viral replication, progressing to viral pneumonia with abnormal pulmonary function tests and progressive ground‐glass opacities on chest CT. In the later stages, immune hyperactivation with widespread hyperinflammation (the ‘cytokine storm’) can escalate to acute respiratory distress syndrome (ARDS). Histological findings in severe COVID‐19 include diffuse alveolar damage, including the more severe form known as acute fibrinoid and organising pneumonia, which is associated with fibrosis and the presence of (micro)thrombi [86]. In an attempt to heal the injury, a chronic immune response with ongoing low‐grade inflammation, immune dysregulation, and airway hyperreactivity [87] may collectively impair functional recovery, contributing to persistent respiratory symptoms.

Lung lesions can persist for up to 12 months posthospitalisation, with unresolved radiological changes in 24% of patients [88]. Common CT findings post‐COVID‐19 include ground‐glass opacities (as in acute COVID‐19), reticulations, consolidations, bronchiectasis, and fibrosis, particularly in mechanically ventilated patients [88, 89]. Importantly, these relate to the presence of respiratory PCC symptoms such as dyspnoea over time [89]. However, most patients with respiratory PCC symptoms have no detectable abnormalities [90]. In rare cases, CT angiography may detect venous thromboembolism (VTE), but the incidence rate of pulmonary embolism—a condition resulting from VTE—has been estimated as <1% in PCC [91]. Single‐cell sequencing of individuals with respiratory PCC and non‐PCC controls from the general community showed a proinflammatory pattern in the small airways, with upregulated neutrophil‐associated activation and increased mucin gene expression in secretory cells [92]. Furthermore, lung‐specific inflammation in PCC has also been observed, revealing PCDH1 (an adhesion molecule modulating airway inflammation) to be associated with cardiorespiratory symptoms [45].

### Immune system

There may also be immune dysregulation in PCC patients, which can occur regardless of the severity of initial COVID‐19 illness [93]. Evidence of ongoing disturbances in the immune response in PCC indicates active inflammation with repair dysregulation rather than solely past tissue damage from acute SARS‐CoV‐2 infection. Multiple aspects of the immune system may be disrupted, including innate and adaptive immune systems. Importantly, emerging data suggest that immunopathology in PCC can differ by biological sex [29]. While males typically experience the more severe acute COVID‐19 and higher mortality, females appear more prone to develop PCC. This discrepancy may be driven by sex‐specific immune pathways, including differential transforming growth factor–β (TGF‐β) signalling and X‐chromosome inactivation processes (e.g. X‐inactive specific transcript [*XIST*] gene expression). For instance, males who later develop PCC can exhibit heightened TGF‐β signalling during acute infection, whereas females on track to develop PCC show reduced *TGFB1* mRNA. Moreover, increased *XIST* in females may alter immune tolerance, potentially raising susceptibility to autoimmune processes. These findings highlight that, although many immunologic aberrations in PCC are shared across sexes, certain pathways may be uniquely modulated, warranting sex‐stratified approaches in future PCC research and therapy [29].

#### Innate immune system

The severity of COVID‐19 is closely linked to the function and regulation of the innate immune system [94], which plays a crucial role in the body's defence against SARS‐CoV‐2 infection by: (1) restricting virus replication in infected cells; (2) recruiting innate immune cells to create an antiviral environment at the site of infection; and (3) activating the adaptive immune response [95]. The efficacy of these responses depends on innate immune cells, particularly myeloid cells and neutrophils. Myeloid cells are key producers of proinflammatory cytokines and exhibit various functional and epigenetic differences post‐COVID‐19. For instance, in severe COVID‐19, hyperresponsive myeloid cells with distinct epigenetic signatures have been observed 1 year postinfection [96]. These epigenetic changes likely contribute to a heightened proinflammatory state, with sustained expression of cytokines and chemokines exacerbating tissue inflammation and impairing resolution of the immune response. Perturbations in myeloid progenitors can contribute to PCC. Specifically, an increase in precursors of the macrophage lineage (CD14^+^CD16^+^ intermediate monocytes) associates with PCC, particularly in cases defined by postacute pulmonary injury [97]. Myeloid cells may also be linked to neurological complications. For example, plasma CCL11 (also known as eotaxin‐1), a chemokine that activates microglia and attracts eosinophils, is elevated in PCC patients suffering from brain fog [98]. Analysis of 657 participants >3 months following hospitalisation with acute COVID‐19 observed elevated myeloid inflammation markers (IL‐1R2, MATN2, CSF3) in PCC patients [45]. In contrast, suppression of myeloid inflammation evidenced by reduced IL‐2 and elevated sCD58 (which is shed by activated myeloid cell during inflammation, thereby disrupting monocyte–lymphocyte interactions) and enhanced tissue repair indicated by elevated markers such as IDS and DNER were associated with fully recovered individuals [45]. These observations suggest that myeloid inflammation and dysfunctional tissue repair mechanisms are key to developing PCC, underscoring the potential of immunomodulatory agents as a promising approach in therapeutic trials (see below).

Neutrophils are known to play a role in the pathogenesis of severe COVID‐19 [99, 100], so it is not surprising that they are also implicated in the pathogenesis of PCC, where neutrophil activation markers can remain persistently elevated [101]. The excessive activation of neutrophils may also enhance the release of neutrophil extracellular traps (NETs) [102], which are often associated with interstitial lung changes and poor pulmonary function [103]. Activated myeloid cells in the lungs, including neutrophils, may contribute to the development of pulmonary fibrosis [104]. A proteomics‐based clustering analysis identified a subset of patients linked to inflammatory immune profiles, indicating neutrophilic activity. In this patient subset, increased neutrophil counts correlated with markers of neutrophil degranulation and NET formation [46].

Finally, a longitudinal study identified localised activation of the complement system as a key factor driving thromboinflammation and hindering recovery after acute COVID‐19 [105]. PCC patients displayed an imbalanced formation of the terminal complement complex (TCC), leading to tissue damage. This persistent immune activation was linked to thromboinflammation, evidenced by elevated markers such as pentraxin 3 and von Willebrand factor, which play roles in promoting clot formation and inflammation. Proteomic studies also revealed the involvement of complement activation in PCC after hospitalised and nonhospitalised COVID‐19 [45, 46]. Elevated markers such as COLEC12 (activator of the alternative complement pathway) and C1QA (a complement degradation product) were associated with PCC, and increased fibrinogen in PCC suggests thrombosis downstream of complement dysregulation, providing a potential therapeutic target [45].

#### Adaptive immune system

In addition to releasing large amounts of proinflammatory cytokines and inflammatory cells, the adaptive immune system generates virus‐specific CD4^+^ T‐cells, CD8^+^ T‐cells, and Bcells, which are crucial for mounting an effective immune response [24, 95]. However, the adaptive immune response can become dysregulated, leading to organ and tissue damage. This immune imbalance may be a significant factor contributing to life‐threatening conditions in COVID‐19 patients [106, 107] and persistent adaptive immune dysregulation is suspected to play a crucial role in PCC. Patients experiencing PCC often exhibit alterations in both cellular and humoral immune mediators, particularly in T‐cell populations [108, 109]. These changes may contribute to ongoing immunopathology, potentially explaining long‐term symptoms such as chronic fever and muscle and joint pain [107, 110]. For instance, specific T‐cell subsets, including CD8^+^‐T‐cells, may remain activated long after the initial infection [111]. This persistent activation might result from virus‐specific responses to residual viral antigens or from polyclonal/bystander activation driven by the chronic inflammatory environment. While virus‐specific activation suggests the presence of lingering viral proteins or RNA fragments, polyclonal or bystander activation could reflect dysregulated immune signalling or the influence of elevated proinflammatory cytokines. Both mechanisms are hypothesised to contribute to the chronic inflammation and tissue damage observed in PCC [111, 112]. Additionally, CD4^+^ effector memory T‐cells may be involved in sustaining this inflammatory environment by continuously providing help to B cells and other immune cells.

The adaptive immune system's dysregulation in PCC is also reflected in altered cytokine and chemokine production. For instance, elevated levels of proinflammatory cytokines like IL‐6, TNFα, and IFNα were observed in the plasma of PCC patients, which may contribute to the chronic inflammatory state [113, 114] and contribute to the ongoing symptoms of PCC, including fatigue and respiratory difficulties [115]. The dysregulation of memory T and B cells in PCC may also contribute to persistent symptoms. Effector memory T‐cells are crucial for providing long‐term protection against pathogens, but in PCC these cells may become dysfunctional due to prolonged antigen stimulation or chronic exposure to proinflammatory cytokines, leading to a state of exhaustion [116]. This dysfunction may impair their ability to respond effectively to new infections or reactivations of latent viruses. Similarly, memory B cells may contribute to the production of autoantibodies through mechanisms such as molecular mimicry or cross‐reactivity, where antibodies generated against SARS‐CoV‐2 antigens inadvertently recognise self‐antigens. This aberrant antibody response can further contribute to the pathology of PCC by promoting autoimmune reactions and tissue damage.

Further underscoring sex‐specific differences in adaptive responses, females with PCC have increased *XIST*, an RNA implicated in autoimmune pathways, while enhanced TGF‐β activity has been noted in males who develop PCC (especially during acute infection) [29]. These patterns suggest that distinct signalling networks may drive aberrant lymphocyte responses in each sex, influencing the pathogenesis and persistence of PCC symptoms.

The interaction between the innate and adaptive immune systems is critical in the development of PCC. Persistent activation of the innate immune system can lead to chronic activation of the adaptive immune system, creating a cycle of ongoing inflammation and tissue damage. For example, dendritic cells can present antigens to T‐cells, perpetuating their activation and contributing to the prolonged immune response [31]. Improper crosstalk between the two arms of the immune system leads to immune dysregulation, inflammation, and clinical symptoms in PCC patients.

#### Autoimmunity

Autoimmunity may play a role in PCC pathophysiology, with new‐onset autoimmune conditions such as systemic lupus erythematosus (SLE) [117] and antineutrophil cytoplasmic autoantibody (ANCA)‐associated vasculitis [118] reported in some patients. The risk of diagnosis with any autoimmune disease within 1 year following COVID‐19 is higher than age‐ and sex‐matched controls without COVID‐19 [119]. Elevated levels of various autoantibodies have been detected in both acute COVID‐19 [120] and PCC [46, 121, 122, 123], which could impair immune function and lead to inflammation and organ damage. For instance, antinuclear/extractable‐nuclear antibodies and elevated TNFα have been observed up to a year after initial COVID‐19, indicating ongoing autoimmune inflammation, which was associated with persisting COVID‐19‐related symptoms [124]. A proteomics study in 97 PCC individuals after mostly mild acute COVID‐19 found enriched autoreactivity in the entire cohort, with >75% displaying reactivity against at least one autoantigen [46]. The exact mechanisms by which SARS‐CoV‐2 triggers autoimmunity in PCC are not fully understood. Hypothesised mechanisms include molecular mimicry between SARS‐CoV‐2 and host antigens, SARS‐CoV‐2‐induced tissue injury causing epitope spreading, or bystander activation [125]. Direct evidence supporting the autoimmunity theory comes from two preprint studies showing that autoantibodies from PCC patients can induce symptoms in animal models [126, 127]. For example, IgG transfer from individuals with PCC to mice induces pain sensitivity, loss of balance and coordination, and muscle weakness, mirroring PCC patient‐reported lingering symptoms [126]. Importantly, different subgroups of PCC patients may experience varying symptoms based on the diversity of autoantibodies [126, 127]. The exact mechanisms by which these autoantibodies induce pathology and symptomatology warrants further investigation, but it appears that autoantibodies associated with neurogenerative proteins (GFAP and NFL) and leukocyte activation markers (EIF5A) may enhance neuroinflammation and neural excitability leading to pain‐associated behaviour, whereas autoantibodies related to skeletal and cardiac muscle pathways (TTN) may impact muscle function leading to impaired movement behaviour rather than directly affecting neural pathways [127].

### Cardiovascular system

Cardiac symptoms represent the third most frequent set of clinical manifestations of PCC [128]. Potential mechanisms for the cardiovascular PCC phenotype include ongoing inflammation, endothelial dysfunction, hypercoagulopathy, autonomic dysfunction, and lasting tissue injury from initial myocardial stress. Systemic hyperinflammation and autoimmunity are implicated in both PCC and cardiovascular events. For example, elevated levels of proinflammatory cytokines in PCC associate with the development of arrhythmias, myocardial infarction, cardiac inflammation, and heart failure [113, 114]. Autoantibodies targeting cardiac‐associated antigens have also been found in PCC patients [129], potentially contributing to cardiovascular events, although they may also arise in response to PCC‐associated tissue damage without being directly pathogenic. Furthermore, acute COVID‐19 is associated with altered coagulation parameters, particularly in severe cases, with elevated D‐dimer and fibrin degradation products correlating with an increased risk of VTE [130]. This hypercoagulable state may persist in PCC, exacerbating the risk of thrombotic events and contributing to the chronic cardiovascular burden. In support, persistent anomalous micro‐clots that were resistant to fibrinolysis were observed in plasma from PCC patients [131]. Autonomic dysfunction, including postural orthostatic tachycardia syndrome (POTS) and orthostatic hypertension, has also been reported long after acute SARS‐CoV‐2 infection [132, 133]. Since the autonomic nervous system regulates cardiovascular functions like heart rate and blood pressure, its dysfunction may contribute to cardiovascular symptoms in PCC.

In more severe cases, particularly among hospitalised patients with prior heart conditions, elevated troponin and abnormalities on echocardiography or cardiac magnetic resonance (CMR) imaging may be present, indicating myocardial injury, myopericardial inflammation and/or fibrosis, or impaired ventricular function [134]. Autopsies of COVID‐19 nonsurvivors revealed multiple cardiac histopathologic changes, including microthrombi and cardiomyocyte necrosis, as well as dense macrophage infiltration in the heart, although without meeting the criteria for myocarditis. The frequent observations of microthrombi and inflammatory infiltrates in fatal cases suggests that similar, although subclinical, cardiac pathology may be present in PCC patients [134]. Direct histopathologic evidence is currently lacking, but CMR imaging shows that even individuals with initially mild COVID‐19 may show ongoing cardiac inflammation, which correlated with ongoing cardiac symptoms [135]. Other imaging findings include persistent myocardial oedema and fibrosis, which may explain persistent functional impairments in these patients [136, 137]. However, the majority of PCC patients experience cardiac symptoms without objective evidence on diagnostic cardiac tests [14, 138].

### Musculoskeletal system

The chronic pain phenotype featuring myalgia and arthralgia can manifest with pathological changes in the musculoskeletal system. Another frequently observed PCC symptom is postexertional malaise (PEM), which is characterised by muscle weakness, fatigue, and pain following mental or physical exertion, although this symptom could not be categorised into a specific PCC phenotype. Although few studies have explored the pathological changes in muscles and joints of PCC patients, emerging evidence points to sustained inflammation, mitochondrial dysfunction, and (micro)vascular damage. Myopathy has been detected through quantitative electromyography in patients with prolonged muscle complaints or fatigue, up to 8 months after mild or moderate COVID‐19 [139]. Muscle biopsies from PCC patients reveal signs of myositis, including muscle fibre atrophy, necrosis, and infiltration of CD68^+^ macrophages and CD3^+^ T‐cells, indicating a disrupted local immune response [140, 141]. One case–control study indicated that exercise‐induced muscle damage followed by regeneration plays a key role in the pathophysiology of PEM [141]. Synovitis has been observed, marked by macrophage and T‐cell infiltration of synovial tissue [142]. This may lead to arthralgia, stiffness, and swelling, resembling conditions like RA. Individuals with preexisting autoimmune conditions such as RA or SLE may experience worsened joint inflammation and damage due to PCC‐induced immune activation.

Given that the musculoskeletal system has relatively high metabolic demands, impaired mitochondrial function has been implicated in symptoms such as reduced muscle endurance and chronic fatigue [93, 143]. Indeed, metabolic and mitochondrial disturbances are exacerbated in PCC patients with PEM, evidenced by decreased oxidative phosphorylation capacity and reduced succinate dehydrogenase activity [141]. Mitochondrial involvement is also indicated by cytochrome c oxidase‐negative fibres, abnormal mitochondrial structure, and subsarcolemmal mitochondria accumulation [140].

COVID‐19‐related vascular damage may also contribute to long‐term musculoskeletal symptoms if blood flow to muscles and joints is chronically impaired [144], and reduced exercise capacity is associated with lower capillarization in skeletal muscle, as well as capillary degeneration and thickened basement membranes in PCC patients [140, 141, 145]. Accumulation of Weibel‐Palade bodies (involved in platelet adhesion) in endothelial cells suggests (micro)thrombosis as a mechanism underlying capillary degeneration [140]. Intramuscular amyloid deposits have also been observed, although these were absent in capillaries, challenging the idea of amyloid‐related vascular blockage [141]. All in all, the array of structural changes in skeletal muscle fibres, ranging from atrophy and immune infiltration to mitochondrial and capillary abnormalities, highlights the musculoskeletal system as a key target in PCC, contributing to symptoms such as muscle fatigue, myalgia, and exercise‐induced malaise.

### Gastrointestinal system

PCC can also affect the gastrointestinal (GI) system, causing symptoms such as abdominal pain, nausea, and diarrhoea in multiple PCC phenotypes [15]. GI PCC symptoms are likely the result of a combination of mechanisms, with viral persistence, immune dysregulation, microbial disturbances, and autonomic dysfunction being the most important. These interconnected processes can lead to inflammation and altered gut motility, manifesting as persistent GI symptoms. The GI tract is a known target of SARS‐CoV‐2 due to the abundance of ACE2 receptors on the brush border of intestinal mucosae [146]. Viral RNA and proteins may persist in the intestines and stool months after acute infection, suggesting that ongoing viral activity in the gut impacts host immune responses underlying GI symptoms in PCC [147, 148, 149, 150]. Indeed, viral antigen persistence in the gut mucosa was associated with PCC symptoms [150].

SARS‐CoV‐2 persistence may drive long‐term immunological activation, with higher anti‐SARS‐CoV‐2 IgG levels in PCC patients compared to vaccination‐matched controls [151], and increased Tcell activation in the guts of PCC patients where SARS‐CoV‐2 RNA was still detectable up to 2 years postinfection [148]. In addition, viral components in stool are linked to persistently high type I interferon levels [152]. Gut inflammation in PCC is also suggested by a proteomic study observing increased DPP10 (a modulator of tissue inflammation that is associated with inflammatory bowel disease) in PCC patients compared to non‐PCC controls [45]. This gut inflammation may cause tissue damage and disrupt normal intestinal function, contributing to GI‐related PCC symptoms [150]. Damage to the gut epithelium may cause increased intestinal permeability, allowing toxins, microbes, and antigens to pass into the bloodstream, triggering systemic inflammation and worsening GI symptoms [153, 154]. Importantly, increased intestinal permeability could also disrupt the BBB, allowing neurotoxic substances and inflammatory markers to enter the brain, potentially leading to neurocognitive symptoms [155]. In acute COVID‐19, research has demonstrated elevated biomarkers of gut barrier integrity, including fatty acid‐binding protein 2, peptidoglycan, and lipopolysaccharide, although such evidence in PCC is currently lacking [156]. Damage to endothelial cells in the blood vessels supplying the intestines may reduce blood flow and cause ischemic changes, with reports of microthrombosis in these vessels contributing to local tissue damage and inflammation [144]. Furthermore, gut inflammation in PCC reduces absorption of the serotonin precursor, tryptophan [152]. As a result, serotonin is depleted, which may hinder vagal nerve activity and thereby disrupt hippocampal functions and memory, providing a potential explanation for neurocognitive symptoms in PCC [152]. Although changes in the microbiome are not directly visible through tissue pathology, PCC patients often exhibit gut dysbiosis [157, 158]. This reduced diversity of commensal microbes (such as *Faecalibacterium prausnitzii* and *Prevotella*) and an overgrowth of potentially pathogenic bacteria—either preexisting or infection‐induced—may further damage the intestinal barrier and perpetuate inflammation, contributing to GI symptoms [157, 159].

### Urinary system

PCC can also involve renal complications, leading to symptoms such as fatigue, peripheral oedema, hypertension, or alterations in urination. In hospitalised COVID‐19 patients, renal manifestations were observed up to 6 months postdischarge, with 35% showing abnormal estimated glomerular filtration rate and 22% experiencing persistent renal dysfunction since the onset of the disease [160]. AKI is a serious complication of COVID‐19 that may contribute to long‐term kidney issues and its incidence increases with disease severity [161]. The histopathological changes associated with COVID‐19‐related AKI are diverse and include both acute glomerular lesions (predominantly collapsing focal segmental glomerulosclerosis and thrombotic microangiopathy) and acute tubular lesions (predominantly acute tubular necrosis), reflecting the multifaceted mechanisms of kidney injury [162]. In COVID‐19, AKI is thought to arise as a severe complication of ARDS requiring intensive care treatment due to a combination of systemic effects [161], or a direct effect of SARS‐CoV‐2 [163]. Indeed, there is evidence that SARS‐CoV‐2 directly infects proximal tubular cells and podocytes leading to cell injury and subsequent tubulo‐interstitial fibrosis, independent of systemic factors like the immune response [163]. Although AKI during the acute phase of COVID‐19 contributes to loss of renal function postinfection and the development of multiple organ system PCC phenotype, deterioration of kidney function has also been observed in nonhospitalised individuals who did not experience AKI during the acute phase [164, 165].

### Systems interaction

**Figure 4:**
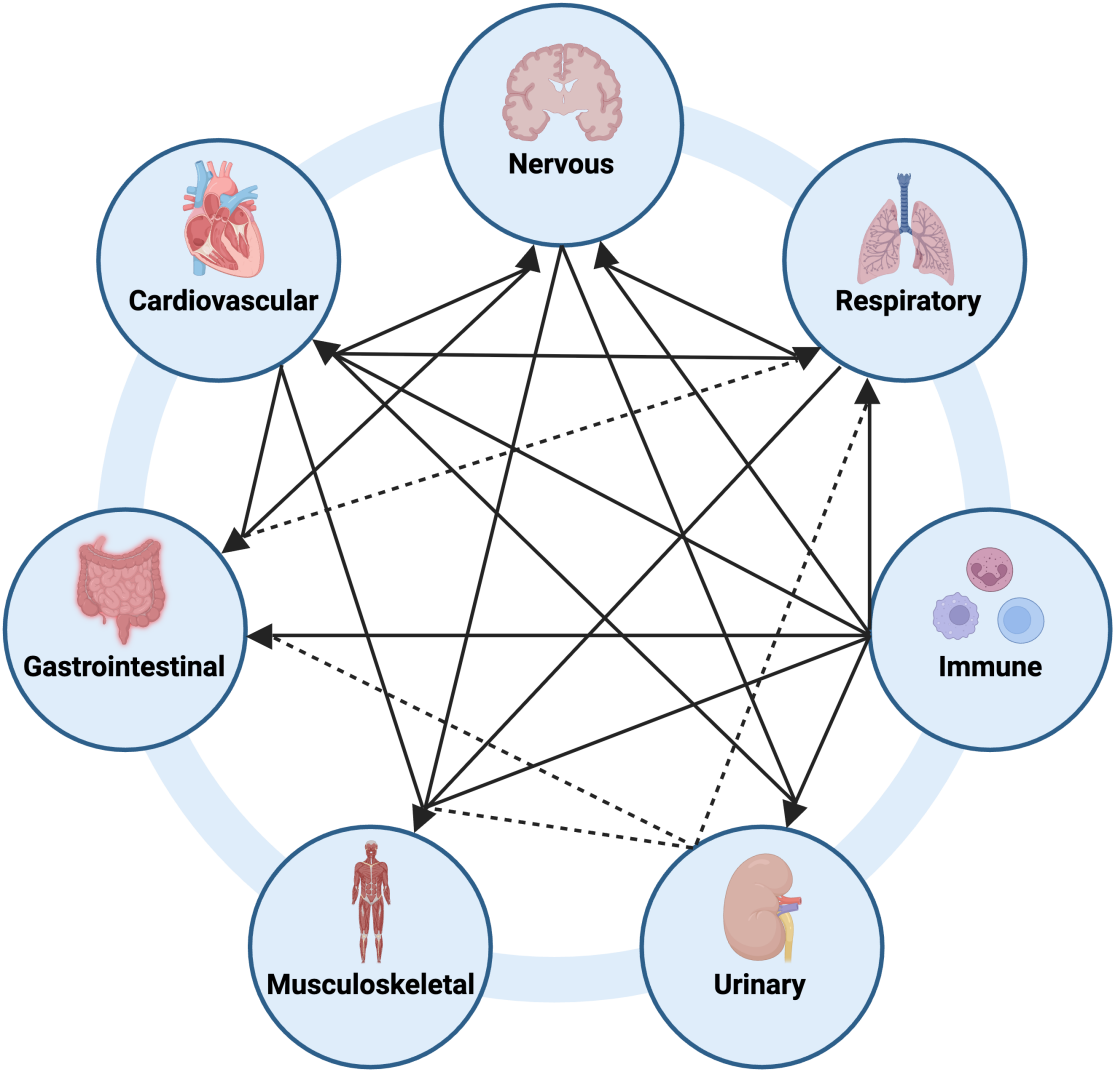
Interactions among systems. This figure illustrates the interconnectivity of major organ systems affected in PCC, highlighting interactions involving the Nervous, Immune, Respiratory, Cardiovascular, Musculoskeletal, Gastrointestinal, and Urinary systems. The Immune system, represented by macrophages, neutrophils, and lymphocytes, mediates systemic inflammation, autoimmunity, and chronic immune activation, potentially impacting all other organ systems. The Nervous system may influence other systems including the Cardiovascular and Respiratory systems through autonomic dysfunction. The Cardiovascular system, affected by endothelial dysfunction and impaired perfusion, subsequently impacts Respiratory (via compromised pulmonary circulation), Urinary (through altered renal perfusion and filtration), and Musculoskeletal systems (due to impaired oxygen and nutrient delivery leading to fatigue and reduced muscular capacity). Gastrointestinal dysbiosis and altered gut permeability directly modulate systemic immune responses. Solid arrows indicate evidence‐supported relationships, while dashed arrows represent hypothesised or less‐characterised links. References are detailed in the main text. This figure was created using BioRender (https://BioRender.com).

The interplay of organ systems in PCC can be conceptualised as a complex network of bidirectional influences modulated by underlying physiological and immunological processes (Figure 4). Persistent immune activation may exert downstream effects on the nervous system, altering autonomic and sensory pathways and thereby influencing cardiovascular regulation and respiratory drive. Similarly, disturbances in respiratory function can compromise oxygen delivery and metabolic efficiency, indirectly affecting muscular strength, exercise tolerance, and gastrointestinal motility. Altered gut microbiota composition and intestinal permeability may in turn feedback into systemic inflammation and impact neurocognitive symptoms. These interconnected pathways underscore the importance of approaching PCC as a multisystem condition, where changes in one system can propagate through various biological axes, ultimately shaping the clinical presentation and prognosis.

## Management

PCC services vary significantly within and across healthcare systems. In the Netherlands, PCC expertise centres have emerged, where personally‐tailored care is provided, and other university hospitals will follow soon. Nevertheless, a fully integrated and comprehensive approach to PCC is lacking in most if not all countries, with most facing a disparity between demand and services provided. Furthermore, limited access to medical centres for often home‐bound patients and reluctance to participate in placebo‐controlled trials (with alternative treatments available outside the study) pose significant challenges to providing adequate PCC care. Ideally, a comprehensive approach should be established to define diagnostic protocols and treatment options based on the latest research, national and international experiences, and expert consensus. To ensure this approach remains up‐to‐date, it should be regularly reviewed to incorporate emerging knowledge and developments.

Ultimately, prevention is key, as no definite treatment exists yet. The most effective way to prevent PCC is by preventing SARS‐CoV‐2 infection through public health measures such as physical distancing, masking, and quarantine when infected. Another key factor is COVID‐19 vaccination, and meta‐analyses have found a preventive effect against PCC (pooled odds ratio ranging from 0.54 to 0.68) [166, 167, 168]. Once PCC is diagnosed, current strategies focus on supportive care to alleviate symptoms and optimise functional performance through physiotherapy, psychological support, cognitive therapy, dietary guidance, and consideration of individual life context (e.g. reasonable workplace adjustments and phased return). Pharmacological management revolves around the use of repurposed drugs, since no approved treatments specific to PCC exist. The safety and efficacy of these treatments have been assessed in clinical trials, often yielding mixed results, with many trials still ongoing. To date, 326 interventional studies related to PCC are currently listed on ClinicalTrials.gov [169] (and see Table 1).

**Table 1:** Overview of (off‐label) drugs for PCC, based on the described pathophysiological mechanisms.

Underlying pathophysiological mechanism	Drug	Proposed mode of action	Trial registry number or reference
**Lingering virus**	**Antivirals**		
	Nirmatrelvir/ritonavir	Protease inhibitor	NCT05595369 NCT05576662
	Favipiravir	Inhibition of RdRp	NCT04448119
	Remdesivir	Inhibition of RdRp	NCT04978259
	**Blood‐derived products**		
	Monoclonal antibodies	Neutralising SARS‐CoV‐2 viral antigens	NCT05013723 NCT05877508 NCT05508295
**Immune dysfunction**	**Antiinflammatories**		
	Naltrexone	Opioid agonist with antiinflammatory actions	NCT05430152 NCT04604704
	Colchicine	Inhibits microtubule polymerisation, with effects on the inflammasome, and reduces neutrophil activation	ISRCTN10665760
	Bezisterim	Proposed antiinflammatory action	[177]
	Corticosteroids (e.g. methylprednisolone)	Inhibition of proinflammatory signals and activation of antiinflammatory signals through glucocorticoid‐mediated effects, e.g. lipocortin‐mediated PLA2 suppression, COX inhibition, inhibition of NF‐κB signalling.	NCT05986422
	**Immunomodulators—biologicals**		
	IL‐1R antagonists (e.g. anakinra)	Inhibition of the proinflammatory action of IL‐1	NCT05926505
	JAK inhibitors (e.g. baricitinib)	Inhibition of JAK/STAT pathway—blocks cytokine signalling pathways to reduce inflammation	NCT06631287
	Tyrosine kinase inhibitors (e.g. imatinib)	Inhibition of signalling pathways that regulate immune cell activation and inflammation	NCT05220280
	TNFα inhibitors (e.g. infliximab)	Inhibition of the proinflammatory cytokine TNFα	NCT05220280
**Gut dysbiosis**	Faecal microbiota transplantation	Restores gut microbiota balance to improve immune function and reduce inflammation	NCT05556733
	Probiotic supplementation	Supports gut health by increasing beneficial bacterial and improving microbial balance	NCT05975034 NCT06643299
**Oxidative stress**	**Antioxidants**		
	Taurine	Scavenging reactive species and maintaining mitochondrial integrity, as well as antiinflammatory actions by inhibiting NF‐κB and multiple proinflammatory cytokines	NCT06721949
	Glutathione, N‐acetylcysteine, and alpha lipoic acid	Scavenging reactive species, regenerating antioxidants, maintaining protein thiol status	NCT05371288
**Thrombosis**	**Anticoagulants**		
	Rivaroxaban	Inhibition of Factor Xa, a key enzyme in the blood clotting cascade	ISRCTN10665760

Abbreviations: IL, interleukin; JAK/STAT, janus kinase/signal transducer and activator of transcription; RdRp, RNA‐dependent RNA polymerase; SARS‐CoV‐2, SARS coronavirus 2; TNFα, tumour necrosis factor alpha.

Current recommendations on pharmacological therapies are mainly symptom‐based [170]. Examples include selective serotonin reuptake inhibitors (SSRIs) for depression and anxiety, nonsteroidal antiinflammatory drugs (NSAIDs) or triptans for headache, benzodiazepines for insomnia, and beta‐blockers for tachycardia and POTS [170]. Repurposed drugs aimed at the suspected underlying mechanisms of PCC are increasingly explored (Table 1). Drugs that target lingering viruses as a putative mechanism include antiviral therapies and neutralising monoclonal antibodies. Other treatments target immune dysfunction, including antiinflammatory drugs, as well as immunomodulatory drugs such as IL‐1 antagonists, JAK inhibitors, and tyrosine kinase inhibitors. Treatments aimed at alternative pathophysiological mechanisms are also being explored, including gut‐focused interventions such as faecal microbiota transplantation or probiotic supplementation for gut dysbiosis, antioxidants for oxidative stress, and anticoagulants for thrombosis. Research focused on underlying mechanisms could pave the way for a personalised treatment approach to PCC, targeting distinct symptom clusters guided by relevant biomarkers. Indeed, patients with specific PCC phenotypes may respond better to tailored, personalised interventions, highlighting the need for clinical trials to refine inclusion criteria. In acute COVID‐19, for example, one study identified a high‐mortality cluster of patients with higher viral loads and shorter symptom duration prior to COVID‐19 testing, with remdesivir showing a significant benefit on mortality in this group [171]. Similar approaches could also be applied to PCC by identifying specific phenotypes that may benefit from tailored treatments, and personalised treatment approaches are highly warranted. However, treatment decisions may also be influenced by other factors contributing to PCC occurrence. For instance, women are disproportionally affected by PCC, and a higher prevalence of depression partly explains this association [18]. This is particularly relevant in the fatigue/neuropsychiatric phenotype, suggesting that addressing mental health, including potential use of antidepressants, should be an important aspect of management. Finally, pharmacological agents may not only be beneficial for treatment, but also in preventing PCC. In particular, early antiviral treatment associated with a reduced risk of developing PCC in observational studies, although validation through randomised controlled trials is awaited [170]. Another drug that may prevent PCC development is metformin, a treatment for type 2 diabetes, which was shown to reduce the risk of PCC by about 41% compared with placebo in a randomised trial of >1,100 individuals [172]. However, this effect is likely independent of its role in diabetes management. Metformin has demonstrated antiviral activity against SARS‐CoV‐2 in cell culture and *ex vivo* in human lung tissue, as well as the ability to mitigate oxidative stress and inflammation. For example, in SARS‐CoV‐2‐infected human bronchial and lung epithelial cell lines, metformin restored autophagy to protect against NLRP3 inflammasome activation and ROS, and inhibited IL‐1β/IL‐1 maturation and NSP6‐induced caspase‐1 cleavage and ROS production [172, 173]. These antiviral, antiinflammatory, and antioxidant mechanisms may reduce the risk of developing PCC.

## Future directions and research gaps

### Current limitations

Despite advances in understanding PCC, several key limitations hinder full elucidation of its pathophysiology and the development of effective treatments. The symptoms of PCC are heterogeneous and identifying subtype‐specific pathophysiological mechanisms remains challenging, complicating the development of standardised diagnostic criteria. Most studies focus on short‐term outcomes, lacking long‐term data to distinguish chronic from relatively acute and temporary symptoms [174]. Biomarker discovery in PCC is still nascent, with inconsistent or nonreproducible findings hampering the development of targeted therapies and disease monitoring [175]. Incomplete understanding of how SARS‐CoV‐2 causes long‐term organ damage further limits progress. Finally, as current research is concentrated in high‐income countries, the influence of factors like ethnicity, socioeconomic status, and access to healthcare has been overlooked. This limitation restricts the generalisability of findings and may lead to underrepresentation of at‐risk populations in research [176].

### Potential areas for future research

**Figure 5:**
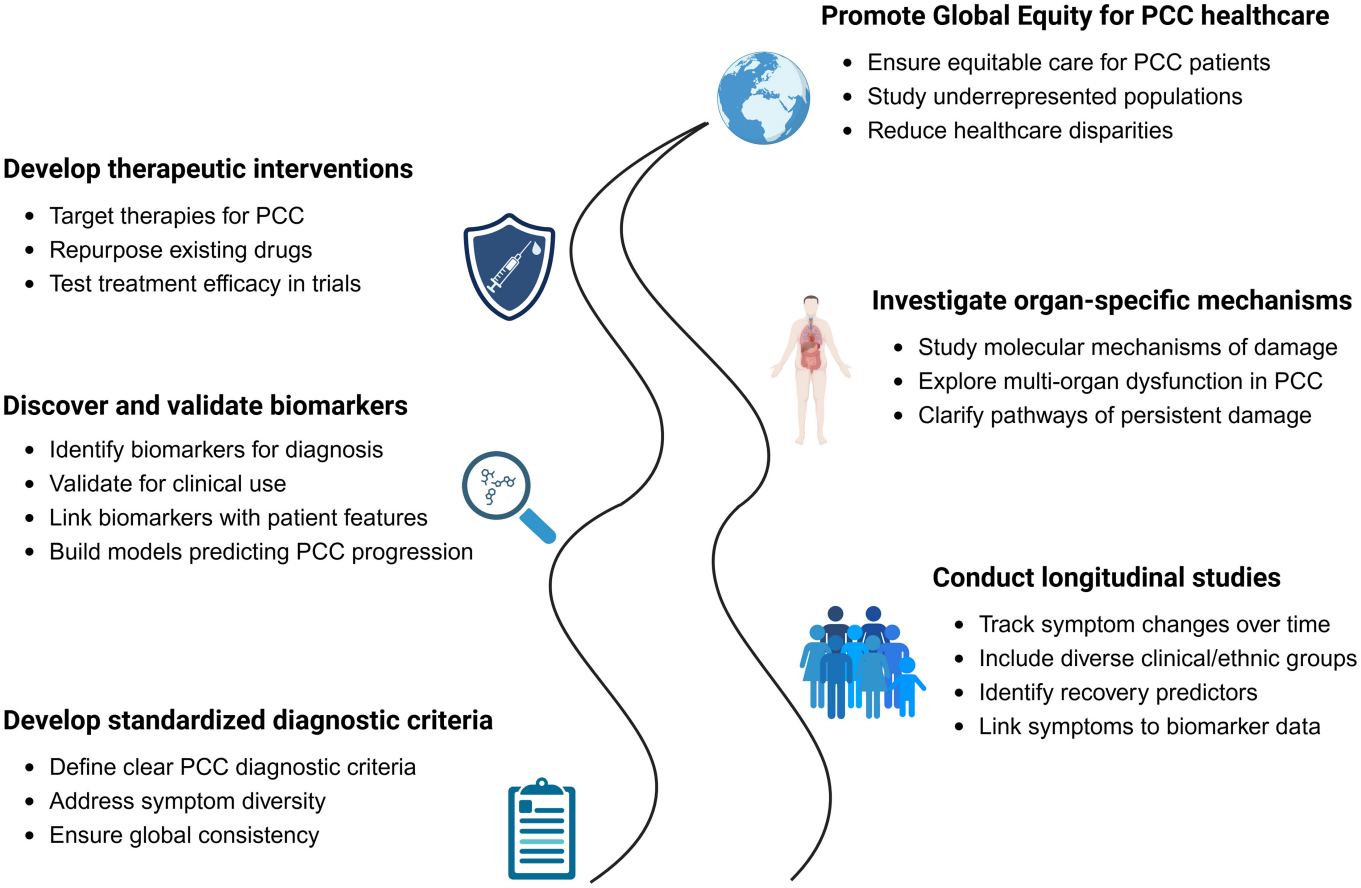
Future directions for advancing PCC research and treatment. This figure was created using BioRender (https://BioRender.com).

Given the current limitations, several key areas warrant further investigation (Figure 5) including: (1) the development of standardised diagnostic criteria through large‐scale studies accounting for symptom diversity; (2) building good registry systems, such as those initiated through PCC expertise centres, to systematically collect and analyse patient data; (3) well‐designed longitudinal studies to track symptoms, complications, and recovery predictors; (4) biomarker discovery and validation for diagnosis, outcome prediction, and treatment monitoring, which requires collaboration across multiple disciplines such as immunology, molecular biology, bioinformatics, and high‐throughput to analyse complex datasets efficiently. Examples include phage‐display immunoprecipitation sequencing to map antibody repertoires and discover potentially relevant immune epitopes, as well as proteomics to identify and quantify circulating biomarkers. In addition, machine‐learning models should integrate patient characteristics and symptomatology (to refine PCC phenotypes) with multi‐omics data to enhance predictive analytics and personalised treatment strategies; (5) mechanistic studies of organ‐specific pathology to investigate the cellular and molecular mechanisms leading to organ damage; (6) therapeutic development, including repurposing existing drugs and the development of novel treatments, with clinical trials across diverse PCC subgroups; and (7) addressing geographic and demographic disparities by focussing on underrepresented populations, particularly in low‐ and middle‐income countries, and understanding how factors like ethnicity and socioeconomic status impact PCC outcomes, thereby helping to develop equitable healthcare strategies for all affected populations.

## Conclusion

PCC is a complex and heterogeneous condition that can affect multiple organ systems and impacts patients' quality of life. The pathophysiology of PCC likely involves a combination of different mechanisms, including viral persistence, immune dysregulation with lingering inflammation, and coagulopathy, among others. Depending on individual risk factors, these pathophysiological mechanisms may produce distinct clinical PCC phenotypes, although complex and overlapping symptom clusters are frequently observed. Nevertheless, characterising and targeting PCC phenotypes with distinct risk factors and pathophysiology, and thus different druggable targets, could pave the way for more personalised treatment approaches. The multifaceted nature of PCC's pathophysiology provides a basis for diverse treatment approaches. Antivirals and immunomodulatory drugs target hallmark pathways such as viral persistence and immune dysregulation, whereas antioxidant, anticoagulant, and probiotic therapies address alternative pathways underlying PCC. Future research should focus on developing standardised diagnostic criteria, identifying reliable biomarkers, and elucidating the underlying mechanisms to guide targeted therapies. A multidisciplinary approach to patient care and continued investigation into potential treatments are essential for improving outcomes for those affected by PCC.

## Author contributions

All authors contributed to the conceptualisation of this review. LEV‐vE and GT designed the outline of the review, wrote the first draft, and prepared the figures. All authors contributed to revision of the article and approved the submitted final version.

## Data Availability

Data sharing not applicable to this article as no datasets were generated or analysed during the current study.
